# Clinical and Financial Implications of Medicine Consumption Patterns at a Leading Referral Hospital in Kenya to Guide Future Planning of Care

**DOI:** 10.3389/fphar.2018.01348

**Published:** 2018-12-10

**Authors:** Patrick M. Kivoto, Mercy Mulaku, Charles Ouma, Alessandra Ferrario, Amanj Kurdi, Brian Godman, Margaret Oluka

**Affiliations:** ^1^Department of Pharmacology and Pharmacognosy, School of Pharmacy, University of Nairobi, Nairobi, Kenya; ^2^Health Commodities and Services Management (HCSM) Program, Management Sciences for Health (MSH), Nairobi, Kenya; ^3^Department of Population Medicine, Harvard Medical School, Harvard Pilgrim Healthcare Institute, Boston, MA, United States; ^4^Strathclyde Institute of Pharmacy and Biomedical Sciences, Strathclyde University, Glasgow, United Kingdom; ^5^Department of Pharmacology, College of Pharmacy, Hawler Medical University, Erbil, Iraq; ^6^Department of Laboratory Medicine, Division of Clinical Pharmacology, Karolinska Institutet, Karolinska University Hospital Huddinge, Stockholm, Sweden; ^7^Health Economics Centre, Management School, University of Liverpool, Liverpool, United Kingdom

**Keywords:** ABC analysis, VEN analysis, medicines, hospitals, expenditure, Kenya

## Abstract

**Background:** Medicines can constitute up to 70% of total health care budgets in developing countries as well as considerable expenditure in hospitals. Inventory management techniques can assist with managing resources efficiently. In Kenyatta National Hospital (KNH), a leading hospital in Kenya, over 30% of expenditure is currently allocated to medicines, and this needs to be optimally managed.

**Objective:** To investigate drug consumption patterns, their costs and morbidity patterns at KNH in recent years.

**Methodology:** Cross-sectional retrospective record review. Inventory control techniques, ABC (Always, Better, and Control), VEN (Vital, Essential, and Non-essential) and ABC-VEN matrix analyses were used to study drug expenditure patterns. Morbidity data was extracted from the Medical Records.

**Results:** Out of an average of 811 medicine types procured annually (ATC 5), 80% were formulary drugs and 20% were non-formulary. Class A medicines constituted 13.2–14.2% of different medicines procured each year but accounted for an average of 80% of total annual drug expenditure. Class B medicines constituted 15.9–17% of all the drugs procured yearly but accounted for 15% of the annual expenditure, whilst Class C medicines constituted 70% of total medicines procured but only 5% of the total expenditure. Vital and Essential medicines consumed the highest percentage of drug expenditure. ABC-VEN categorization showed that an average of 31% of medicine types consumed an average of 85% of total drug expenditure. Therapeutic category and Morbidity patterns analysis showed a mismatch between drug expenditure and morbidity patterns in over 85% of the categories.

**Conclusion:** Class A medicines are few but consume the largest proportion of hospital drug expenditure. Vital and essential items account for the highest drug expenditure, and need to be carefully managed. ABC-VEN categorization identified medicines where major savings could potentially be made helped by Therapeutic category and Morbidity pattern analysis. There was a high percentage of non-formulary items, which needs to be addressed. Inventory control techniques should be applied routinely to optimize medicine use within available budgets especially in low and middle income countries.

## Introduction

Medicines have been used across countries to alleviate patients’ suffering and improve their lives with an agreed list of essential medicines to reduce morbidity and mortality (Hogerzeil et al., 2013; Wirtz et al., 2017). Medicines are now typically seen as one of the most cost-effective interventions to improve health especially as more standard medicines lose their patent and become available as low cost generics or biosimilars (Simoens, 2012; Godman et al., 2014b, 2017a; Moorkens et al., 2017). However, the inappropriate use of medicines can appreciably increase costs through adverse drug reactions (ADRs), drug-drug interactions, and wasted resources, compromising the quality of care and increasing mortality (WHO, 2002; Davies et al., 2009; Wu et al., 2010; Godman et al., 2013; Dechanont et al., 2014; Mouton et al., 2015; Kiguba et al., 2017).

Overall, medicines constitute an appreciable component of total health care costs (Clemente et al., 2008; Lu et al., 2011), comprising up to 20–40% of health care budgets in many developing countries (Cameron et al., 2009; Lu et al., 2011). This may increase up to 70% in low and low-middle income countries (LMICs) (mean of 27.6–30.4%), where there are also appreciable co-payments (Cameron et al., 2009; Lu et al., 2011; Ofori-Asenso and Agyeman, 2016; Nguyen et al., 2015). Co-payments can account for up to 76.9% of total pharmaceutical expenditure, which can potentially be catastrophic for the family in terms of purchasing essentials such as food alongside medicines if family members become seriously ill (Cameron et al., 2009; Nguyen et al., 2015). For instance in Kenya, even the cost of one radiotherapy session at US$5 – 10 for patients with cancer in the major public hospital can be prohibitively expensive for disadvantaged Kenyans who have to pay this as they typically live on US$1 per day or less, and the estimated costs of treating patients with cancer of up to US$5000/patient is just unaffordable for the majority of patients and their families (Mbui et al., 2017; Osman, 2017; Atieno et al., 2018). Schemes have now been launched by pharmaceutical companies in Kenya to increase access to essential medicines for patients with cardiovascular diseases given current concerns with affordability and outcomes (Sandoz, 2015; Mbui et al., 2017).

Within hospitals, the appropriate use of medicines, and their availability, is vital to improve the care of patients within available resources aiding early discharge. The irrational use of use of medicines in hospitals can lead to a number of problems including reduced access to essential medicines, reduced patient attendance rates due to stock outs and loss of patient confidence in the healthcare system, as well as increasing ADRs and length of stay (WHO, 2002; Davies et al., 2009; Holloway and van Dijk, 2011; Kiguba et al., 2017). Health institutions should aim at stocking an agreed range of medicines along with initiatives to improve their use and the supply chain to address concerns (Meyer et al., 2017). Essential medicines are defined by the World Health Organization (WHO) as “*those that satisfy the priority health care needs of the population*”, and are selected “*with due regard to public health relevance, evidence on efficacy and safety, and comparative cost-effectiveness*” (WHO, 2017b). Selecting such medicines across countries typically begins with defining a list of common diseases for each level of healthcare; subsequently selecting first and second line treatment choices for hospital formularies and Standard Treatment Guidelines (STG). This has been exemplified for instance in Scotland with their Regional formularies and in Sweden with their regional formularies such as the ‘Wise List’ in Stockholm County Council (Gustafsson et al., 2011; Bjorkhem-Bergman et al., 2013).

Improved management of medicines in hospitals should lead to improved medicines availability thereby improving patient outcomes and reducing morbidity and mortality (Pillans et al., 1992). In view of this, pharmaceutical stores need to be planned, designed, organized and maintained in a manner that results in efficient clinical and administrative services (Khurana et al., 2013; Meyer et al., 2017).

Methods used to identify irrational medicine use include instigating drug utilization studies using the Anatomical Therapeutic Chemical/Defined Daily Dose (ATC/DDD) methodologies, Drug Use Evaluation (DUE) and qualitative methods to assess the extent of current utilization patterns and any concerns (WHO, 2003; Godman et al., 2016). This includes aggregate data methods such as ABC analysis, Therapeutic Category (TC) analysis and VEN analysis, which is an inventory categorization method whereby medicines are classified according to their perceived public health impact into vital, essential and non-essential. These methods are used by Drug and Therapeutic Committees (DTCs), particularly in LMIC countries, to better manage their formulary lists and identify gaps in drug use (Laing et al., 2001; Kumar and Chakravarty, 2015). ABC analysis groups medicines based on their cumulative cost percentage. Class A medicines constitute 10–20% of the medicines within hospitals but typically account for 70–80% of the total drug budget; Class B medicines comprise the next 10–20% of available medicines but consume 15–20% of the drug budget; with Class C medicines constituting the remaining 60–80% of medicines in the facility but accounting for only 5–10% of the annual drug budget (Quick et al., 1997; Holloway and Green, 2003; Management Sciences for Health and World Health Organization, 2007; WHO, 2012). VEN analysis (Vital – V, Essential – E, and Non-Essential – N) subsequently, as mentioned, classifies medicines based on their perceived public health impact. Vital medicines (V) are potentially life-saving, have significant side-effects or have major public health importance. Essential medicines (E) are effective against less severe but significant forms of disease, but are not absolutely vital to providing basic healthcare. Non-essential medicines (N) are used for minor or self-limiting illnesses, are of questionable efficacy and typically have a high cost for marginal therapeutic gain (Management Sciences for Health and World Health Organization, 2007; WHO, 2012). Such analyses have worked well in LMICs including areas such as cancer, which are a growing priority with increasing prevalence rates as well as increasing costs of medicines (Godman et al., 2017b; Atieno et al., 2018; Jakupi et al., 2018).

No institution in LMIC countries typically has adequate funds to fully procure all suggested medicines on the formulary list, especially with medicines already typically accounting for a third of total hospital expenditure, which is generally the case in tertiary hospitals in these countries (Kumar and Chakravarty, 2015). This requires prudent selection of medicines that is evidence based, coupled with setting priorities that will enable institutions to improve their overall efficiency of medicine use given ongoing concerns with medicine availability and their costs (WHO, 1999; Kumar and Chakravarty, 2015). ABC-VEN analyses may be employed in hospitals to enhance future selection and stock management (Quick et al., 1997; Thawani et al., 2004; Ramanathan, 2006; Gupta et al., 2007; Devnani et al., 2010; Anand et al., 2013; Jakupi et al., 2018).

However, accurate knowledge of medicine use and priorities among hospitals in Kenya is still inadequate. Consequently, the objectives of this study were to determine and compare medicine classes that accounted for the greatest proportion of the drug budget in a leading tertiary hospital in Kenya, Kenyatta National Hospital (KNH); secondly, determine and compare medicine use according to their potential health affects (VEN), and lastly to rationalize the therapeutic categories of medicine use. The findings will be used to guide future interventions in this hospital and wider, with the goal of improving the rational use of medicines, with KNH setting the trend for other hospitals in Kenya through its training and comprehensive services. As such, seek to maximize patient outcomes within available resources. We believe this is the first time that such an extensive analysis has been undertaken in Kenya, providing guidance to other hospital facilities in Kenya and wider among secondary and tertiary hospitals across Africa.

## Methodology

### Study Design

The ABC analysis was conducted as a retrospective cross sectional record review. For each year of the study (2013–2015), annual consumption data along with the related expenditure incurred on each item was retrieved from the pharmaceutical stores of KNH, which are located on the ground floor of the hospital. Data was subsequently transferred into the ABC Analyzer 5, 80/20 Analytics (a software under development and license number granted 8579574233285627).

The ABC analyzer grouped the listed medicines into the three categories – A, B, and C, based on the cumulative cost percentage of 80, 15, and 5%, respectively. The VEN analysis was a descriptive retrospective study. The VEN status of each medicine was obtained from the KNH formulary and the Kenya Essential Medicines List (KEML), both of which were developed by a multidisciplinary team of key specialists including physicians, surgeons, pediatricians, and pharmacists (Quick et al., 1997; The Ministry of Medical Services and Ministry of Public Health & Sanitation Kenya, 2010; Ministry of Health Republic of Kenya, 2016).

After performing the ABC analysis, the medicines were assigned to a therapeutic category to develop a morbidity pattern for drug use. This was based on the KNH formulary, the World Health Organization (WHO) model list of Essential medicines, the Anatomical Therapeutic Chemical (ATC) codes (WHO, 2013) and the International classification of diseases (ICD-10) developed by the WHO. In addition, the annual morbidity data for the years 2013–2015 was extracted from KNH’s Health information database and entered into an MS Excel spreadsheet for analysis.

### Study Site

The study was conducted at KNH which is a 2000 bed national teaching and referral hospital in Kenya with an annual average of 70,000 inpatients and 500,000 outpatients. KNH is the largest public referral hospital in the region, offering quality specialized healthcare to patients from across Kenya, Great lakes region, Southern and Central Africa (Kenyatta National Hospital, 2014). KNH also offers most of the medical specialty and related services including specialized surgeries such as open heart surgery, neurosurgery, critical care services, oncology, burns management and renal services (including kidney transplantation). KNH also launched its formulary in September 2013 with the support of Management Sciences for Health (MSH) (Otieno, 2013), providing guidance to other hospitals in Kenya.

Kenyatta National Hospital has a Supply Chain department which is responsible for the purchase, storage of medicines, and other medical supplies, across the hospital. There are also a number of donor funded programs that are also handled by the Supply Chain department including HIV/AIDS and Malaria programs. Most of the records in the Supply Chain department are manual but as from 2014 a Health Management Information System (HMIS) was launched; however, it is still not fully operational. The procurement of medicines follows the government system of tendering and the lowest bidder wins the tender.

### Sources of Data

The data sources included S3 cards (Stores Ledger and Stock Control card), S5 cards (Bin cards, records stock movement), S13 cards (Counter receipt voucher cards), S11 (Issue voucher), the security receiving book as well as HMIS and any other relevant records that could provide pertinent consumption data, prices and annual morbidity.

### Inclusion and Exclusion Criteria

The study included the drug procurement records for the years 2013–2015. Records for medicines procured directly under the Pharmacy budget were included. The study also included records of any medicines borrowed, donated or returned to the store. S11 were used for medicines borrowed and S13 for medicines donated.

The study excluded medical gasses, mainly oxygen and nitrogen, dialysis solutions and some dressing for burns that were not procured under the pharmacy budget. The study also excluded records of medicines kept at the Private Wing Store, which are procured independently of the Main Hospital, as we just wanted to concentrate on the public health system where the majority of patients are treated. For the TC and morbidity data, the study excluded ICD-10 classes whose annual morbidity data were missing. ICD-10 classes which did not have identifiable medicines were also excluded from the study.

### Sample Size and Sampling Method

A universal sampling technique was used whereby every record with relevant information to the study was included. A sample size determination was not conducted for the ABC, VEN, and TC analysis since this was an annual expenditure study, and every record was included in the analysis to obtain the most accurate expenditure and consumption data (medicine type by ATC class as opposed to utilization broken down by defined daily doses) as possible. This is in line with previous publications (Migbaru et al., 2009; Anand et al., 2013; Kumar and Chakravarty, 2015; Kastanioti et al., 2016; Mousnad et al., 2016).

### Data Collection Procedures

A data collection tool was adapted from WHO studies for the ABC and TC analysis (Holloway and Green, 2003; Rankin, 2012). The relevant information included the drug code, drug name, pharmaceutical formulation, unit of issue, quantity and unit price. Data for the VEN categorization were obtained from the KNH formulary and the KEML (The Ministry of Medical Services and Ministry of Public Health & Sanitation Kenya, 2010; Otieno, 2013; Ministry of Health Republic of Kenya, 2016). The morbidity data was extracted and entered onto a predesigned data collection form as per the WHO- ICD-10 system for the years 2013 to 2015 by a research assistant trained on the use of the data extraction forms.

### Study Variables and Definitions

For the ABC analysis, the outcome variables of interest were the number of medicine types (ATC 5) belonging to the A, B, and C classes and their percentage annual expenditure. The ABC-VEN matrix also categorizes medicines into three categories of interest (Category I, II, and III). Category I comprised of medicines in the AV, AE, AN, BV, and CV categories. Category II comprised of medicines in the BE, BN, and CE categories, and Category III in the remaining CN category, i.e., the first letter is from the ABC analysis and the second letter from the VEN analysis. For the TC and Morbidity analysis, the main outcome variable was the proportion of expenditure that matched the morbidity patterns. Annual morbidity data was entered into the health information system (HIS) by the Medical Record staff at the Health Information Department using the ICD-10 system. The data entered on a daily basis was aggregated annually to give the number of cases of each disease encountered in the hospital for the whole year.

### Quality Assurance and Data Management

Double manual data entry was performed as part of quality assurance. Firstly onto the predesigned data collection tool and subsequently onto the MS Excel designed form. This approach was undertaken to improve the accuracy of the data for analysis. Verification of the data was undertaken to ensure the collected and recorded data was accurate by randomly sampling the entries and cross checking them with the source documents. The data collected were cleaned by cross checking, which included removing errors such as double entries and misplaced information. A daily backup was undertaken using a flash disk which was password protected. All the backups were stored under lock and key with only the researcher having access to the keys. The researcher verified all the information entered in the Microsoft Excel worksheet on a daily basis to ensure correct entries. A pilot study was performed beforehand to ensure the data collection forms fully captured all the information required.

### Data Analysis

As mentioned, the ABC analysis was conducted using the ABC analyzer 5, 80/20 Analytics (a software under development and license number granted 8579574233285627). The data was subsequently transcribed onto an MS Excel spreadsheet for quantitative analysis. The statistical analysis was carried out using MS Excel statistical functions. The annual expenditure of individual items was calculated by multiplying the annual drug consumption by the unit price (Consumption × Cost) and arranged in descending order. The percentage of annual drug expenditure and cumulative drug expenditure percentages were subsequently calculated. The VEN classification of the medicines was based on the KNH formulary and the KEML. The medicines were classified using a pre-designed form and the total percentage of expenditure for each category calculated.

For the ABC-VEN matrix analysis, a comparison of the ABC analysis with the VEN classification was subsequently undertaken to populate the ABC-VEN matrix, which comprised the three categories, namely Category I, II, and III.

Expenditure for each category was calculated from the ABC analysis, with the data subsequently transferred onto an Excel spreadsheet for quantitative analysis. Each ICD-10 class was matched with the expenditure on medicines for that class. The cumulative cost of the medicines in each category was calculated and the percentage of total expenditure for each year also calculated. Utilization was based on each drug name (ATC code) and the units of issue. We used a conversion rate of USD 1 = KES 101.3 (Central bank of Kenya^1^) to convert drug expenditure data from Kenya shillings to United States Dollars in line with recent publications (Atieno et al., 2018).

### Ethics Statement

Ethical approval was sought from the KNH-UoN Ethics and Research Committee to conduct the ABC, VEN, TC analysis and approval was received in February 2016, approval number P668/10/2015. Since the Pharmaceutical store is under Supplies department, the records were assessed after getting authority.

## Results

### Annual Consumption and Expenditure on Drugs at KNH

A total of 812 different medicines (ATC 5) were procured in 2013 and 811 in 2014 and 2015 of which 652 (80%) were in the formulary and 159 (20%) were non-formulary. The total number of different medicines procured and their expenditure for the period 2013–2015 is shown in Table 1.

**Table 1:** Annual expenditures on medicines at KNH drug store for 2013–2015.

Year	Total number of medicine types procured	Drug expenditure in Kshs	Total expenditure in USD$	% of Total annual hospital expenditure
2013	812	400,625,444.17	3,954,841.5	33%
2014	811	406,391,886.87	4,011,765.9	33%
2015	811	452,064,244.35	4,462,628.3	34%
Total		1,259,081,575.39	12,429,235.7	


### ABC Analysis

Class A drugs represented 107 (13.2%), 110 (13.6%) and 115 (14.2%) of the total medicines analyzed for the years 2013, 2014, and 2015, respectively. Class A medicines consumed the largest proportion of the total budget at 79.9% for 2013 and 2014 and 79.8% for 2015. Class C medicines represented the highest number of different medicines at 576 (70.9%), 566(69.8%), and 558 (68.8%) for 2013, 2014, and 2015, respectively. These class C medicines consumed only an average of 5% of the total budget. The trend for 3 years was similar as shown in Table 2.

**Table 2:** ABC Analysis of the different medicine types at the KNH drug store for the period 2013–2015.

Analysis parameter	*N* (%) of medicine types procured			% Total annual hospital expenditure on medicines		
		
	2013	2014	2015	2013	2014	2015
A	107 (13.2)	110 (13.6)	115 (14.2)	79.9	79.9	79.8
B	129 (15.9)	135 (16.6)	138 (17)	15.1	15	15.1
C	576 (70.9)	566 (69.8)	558 (68.8)	5	5.1	5.1
Total	812	811	811	100	100	100


From the ABC analysis, the top 10 different medicines for each year and their units of issue from 2013 to 2015 were identified (Table 3).

**Table 3:** Top 10 medicines from ABC analysis and their expenditure for KNH drug store 2013–2015.

Item code	Item description and ATC code (where known)	Unit of issue	2013			2014			2015		
					
			Quantity	Unit price USD	Unit price Kshs	Quantity	Unit price USD	Unit price Kshs	Quantity	Unit price USD	Unit price Kshs
SS001	Human Albumin -20% Solution (B05AA01)	100 ml bottle	2540	67.13	6,800.00	2730	51.83	5,250.00	1907	51.83	5250.00
SG001A	Inj Acyclovir 250 mg (J05AB01)	Amp	9900	15.59	1579.5	13555	14.60	1.479.40	19470	13.70	1388.00
SE053	InJ Heparin sodium 5000 IU/ml (B01AB01)	5 ml Vial	48600	3.02	305.99	45503	2.86	290.03	44100	1.48	150.00
SS025	Inj Na chloride 0.9% solution (B05XAO3)	500 ml bottle	304149	0.41	42	303562	0.42	43	151480	0.41	42.00
SC044	Inj Phenytoin Na, 50 mg/ml (N03AB02)	5 ml Amp	41460	2.36	238.6	32465	2.42	245.02	33275	2.42	245.02
SE051	Inj Enoxaparin 100 mg/ml (B01AB05)	0.4 ml syringe				39250	2.63	266.89	63200	2.80	284.00
SA027A	Inj Cisatracurium 2 mg/ml (M03AC11)	10 ml Amp	5600	14.14	1,432.70	4945	14.14	1.432.70	5170	14.14	1432.70
SS035	Injectable three chamber bag	1,000 ml bag	2010	38.50	3,900.00	2156	37.76	3825.45			
SF059	Inj Meropenem 1 gm (J01DH02)	Vial	13800	5.41	547.76	17757	6.28	636.11	14440	4.15	420.00
SH033	Inj GCSF, 30 miu (L03AA02)	Prefilled syr	820	85.88	8,700.00						
SS049	Triple chamber parenteral nutrition	2000 ml bag								67.92	6880.00
SF027A	Inj Ceftazidime 2 g (J01DD02)	Vial				3315	19.25	1,950.00			
SA028	Isoflurane -liquid for inhalation (N01AB06)	250 ml bottle	1298	50.35	5,100.00				4350	40.05	4057.39


The results show that there is a decrease in expenditure for sodium chloride infusion, heparin injections and recombinant granulocyte colony stimulating factor (G-CSF) over the 3 years, whilst there was an increase in expenditure on meropenem, acyclovir and isoflurane from 2013 to 2015. The Injection three chamber bag was overtaken in expenditure by triple chamber parenteral nutrition in 2015. These bags contain three components namely: glucose 19%, amino acid combinations and intra lipids (20%), which are reconstituted for total parenteral nutrition.

### VEN Analysis

The VEN analysis showed that Vital items (V) accounted for an average of 22.8% (185) of the different medicine types procured over the 3 years, Essential items (E) for 53.3% (432) and Non-essential items accounted for 23.9% (194) the different medicine types procured (Table 4). The findings also revealed that there were a number of non-formulary (NF) items procured and consumed in the hospital during the 3 years, and these accounted for an average of 17.4% (141) of all medicine types procured. Human normal immunoglobulin 5% was the most expensive non-formulary medicine procured during the last 2 years of the study period. It accounted for 0.9% of total expenditure in 2014, increasing to 3% of total expenditure in 2015.

**Table 4:** VEN analysis of medicines at KNH drug store for the period 2013–2015.

Analysis parameter	*N* (%) of total medicine types procured			% Annual expenditure on medicines		
		
	2013	2014	2015	2013	2014	2015
V	177 (21.8)	201 (24.8%)	178 (21.9)	37.7	36.3	27.3
E	433 (53.3)	433 (53.4)	431 (53.1)	56.8	57.6	61
N	202 (24.9)	177 (21.8)	202 (24.9)	5.5	6.1	11.8
Total	812 (100)	811 (100)	811 (100)	100	100	100


The percentage annual expenditure on medicines is based on the annual drug expenditure shown in Table 1.

### ABC-VEN Matrix Analysis

Results of the ABC-VEN matrix analysis for KNH from 2013–2015 are shown in Table 5. The percentage annual expenditure on medicines is again based on the annual drug expenditure shown in Table 1.

**Table 5:** ABC-VEN matrix analysis of medicines procured at KNH drug store for 2013–2015.

Analysis parameter	*N* (%) of total medicine types procured			% Annual expenditure on medicines		
		
	2013	2014	2015	2013	2014	2015
AV	37 (4.6)	38 (4.7)	34 (4.2)	32.6	31.5	22.1
AE	64 (7.9)	66 (8.0)	70 (8.6)	44.7	45.9	49.9
AN	6 (0.7)	6 (0.7)	11 (1.4)	2.6	2.5	7.9
BV	34 (4.2)	33 (4.1)	35 (4.3)	4.0	3.7	3.9
BE	81 (10)	81 (10)	75 (9.3)	9.2	9.1	8.3
BN	15 (1.8)	21 (2.6)	28 (3.5)	2.0	2.3	2.9
CV	106 (13.1)	106 (13.1)	109 (13.4)	1.1	1.1	1.3
CE	288 (35.5)	286 (35.3)	286 (35.3)	2.9	2.6	2.8
CN	181 (22.3)	174 (21.5)	163 (20.1)	0.9	1.3	1.0
Total	812 (100)	811 (100)	811 (100)	100	100	100


The results showed that the highest costing medicines, which are also in the Vital and Essential category, consumed the highest expenditure on medicines. On average, AV drugs accounted for 36 (4.5%) of the different medicine types consumed but 28.7% of total expenditure on medicines, with AE drugs accounting for on average 67 (8.2%) of the different medicines consumed but 46.8% of the total expenditure on medicines (Table 5). The cheaper and non-essential medicines (CN class) consumed on average only 1% of the total expenditure on medicines.

ABC-VEN categorization revealed that 247 (30.4%), 249 (30.7%), and 259 (31.9%) medicines belonged to Category I for 2013, 2014, and 2015, respectively, and consumed approximately 85% of total annual expenditure on medicines (Table 6). Category II had the majority of the items and consumed an average amount of total drug expenditure, with Category III items consuming only an average of 1% of the total medicine expenditure over the 3 years (Table 6).

**Table 6:** ABC-VEN Matrix categorization for medicines at the KNH drug store for 2013–2015.

Analysis parameter	*N* (%) of total medicine types procured			% Annual expenditure on medicines		
		
	2013	2014	2015	2013	2014	2015
Category I	247 (30.4)	249 (30.7)	259 (31.9)	85.0	84.7	85.0
Category II	384 (47.3)	388 (47.8)	389 (48.0)	14.1	14.0	14.0
Category III	181 (22.3)	174 (21.5)	163 (20.1)	0.9	1.3	1.0
Total	812 (100)	811 (100)	811 (100)	100	100	100


### Therapeutic Category and Morbidity Pattern Analysis

The annual morbidity for KNH is shown in Table 7. The morbidity data for 2013 were only partially available; consequently this is not presented in Table 7. The annual morbidity data in descending order for 2014 and 2015 shows almost a similar pattern with ICD-10 class S00-T99 (Injuries, poisoning and certain other consequences of external cause, Burns) having the highest number of cases and ICD-10 class H00-H59 (Diseases of the eye and Adnexa) having the least number of cases.

**Table 7:** Morbidity pattern at KNH for 2014–2015.

ICD-10 code	Disease	2014	2015
			
		*n* (%)	*n* (%)
S00-T99	Injuries, poisoning and certain other consequences of external causes, Burns	7274 (15.0)	6958 (14.5)
I00-I99	Diseases of the Circulatory system	5306 (10.9)	5305 (11.0)
A00-B99	Certain infectious and parasitic diseases	4815 (9.9)	5018 (10.4)
C00-D48	Neoplasms	4800 (9.9)	5253 (10.9)
J00-J99	Diseases of the Respiratory system	4774 (9.8)	4818 (10.0)
N00-N99	Diseases of the Genitourinary system	3533 (7.3)	3831 (8.0)
K00-K95	Diseases of the Digestive system	2988 (6.2)	2920 (6.1)
E00-E89	Endocrine, Nutritional and metabolic disorders	2818 (5.8)	2840 (5.9)
G00-G99	Diseases of the Nervous system	1269 (2.6)	1324 (2.8)
M00-M99	Diseases of the Musculoskeletal system and connective tissue	754 (1.6)	844 (1.8)
L00-L99	Diseases of the Skin and subcutaneous tissue	711 (1.5)	690 (1.4)
D50-D59	Diseases of the blood and blood forming organs and certain disorders involving the immune system	492 (1.0)	503 (1.0)
F00-F99	Mental, Behavioral disorders	368 (0.8)	362 (0.8)
H00-H59	Disease of the Eye and Adnexa	314 (0.6)	354 (0.7)
**Total**		**48484** (100)	**48137** (100)


**FIGURE 1:**
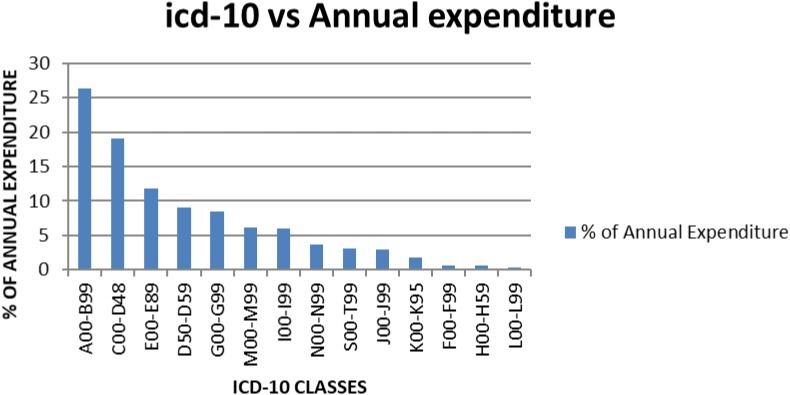
Average annual expenditure for KNH according to the ICD-10 classes for the year 2013–2015.

Average morbidity data patterns from 2013 to 2015, and average annual consumption expenditure on medicines, was computed from the ABC analysis data and tabulated as shown in Table 8 and Figure 1.

**Table 8:** Average morbidity patterns and drug expenditure data for the years 2013–2015 at KNH.

ICD-10 code	Disease	Average *N* (%)	% Average Annual drug expenditure
S00-T99	Injuries, poisoning and certain other consequences of external causes, Burns	5698 (14.9)	3.1
I00-I99	Diseases of the Circulatory system	4303 (11.2)	6
C00-D48	Neoplasms	3989 (10.4)	19
A00-B99	Certain infectious and parasitic diseases	3969 (10.4)	26.3
J00-J99	Diseases of the Respiratory system	3741 (9.8)	2.9
N00-N99	Diseases of the Genitourinary system	2900 (7.6)	3.7
K00-K95	Diseases of the Digestive system	2340 (6.1)	1.8
E00-E89	Endocrine, Nutritional and metabolic disorders	2250 (5.9)	11.8
G00-G99	Diseases of the Nervous system	1165 (3.0)	8.5
M00-M99	Diseases of the Musculoskeletal system and connective tissue	654 (1.7)	6.2
L00-L99	Diseases of the Skin and subcutaneous tissue	562 (1.5)	0.3
D50-D59	Diseases of the blood and blood forming organs and certain disorders involving the immune system	383 (1.0)	9.1
F00-F99	Mental, Behavioral disorders	285 (0.7)	0.6
H00-H59	Disease of the Eye and Adnexa	278 (0.7)	0.6
**Total**		**38292**	**100**


When the percentage annual number of cases and annual drug consumption expenditure was calculated, the results showed that certain infectious and parasitic diseases (ICD-10 class A00-B99) had the highest annual drug expenditure (26.3%), with medicines for neoplasms the second highest expenditure (19%) (Table 8). Diseases of the skin and subcutaneous tissue (ICD-10 class L00-L99) had the lowest annual drug expenditure (0.3%). The highest number of cases seen in KNH was, as mentioned, in the ICD-10 class S00-T99 (Injuries, poisoning and certain other consequences of externals causes, burns).

Two ICD-10 classes showed almost a similar annual number of cases and annual drug expenditure, i.e., ICD-10 class F00-F99 (Mental, behavioral disorders) and H00-H59 (Disease of the eye and Adnexa) at 0.7 and 0.6%, respectively. Some ICD-10 classes had a higher annual drug expenditure compared to the annual number of cases, i.e., ICD-10 class I00-I99 (Diseases of circulatory system) and S00-T99 (Injuries, poisoning and certain other consequences of external causes, Burns) at 6 and 11.2% for the number of cases seen and 13.1 and 14.9% for drug expenditure, respectively. Annual drug expenditure is again based on the figures in Table 1.

Further analysis of the expenditure on medicines based on the KNH Formulary showed an increase in expenditure in 2015 for anesthetic and theater agents and Immunologicals, and a decrease in expenditure on plasma substitutes (Table 9).

**Table 9:** Expenditure on medicines as per the KNH formulary therapeutic categories for 2013–2015.

NO	Therapeutic category	Total expenditure (Kshs)		
		
		2013	2014	2015
(1)	Anesthetic and theater agents	31,847,419.50	29,882,455.25	44,372,410.89
(2)	Analgesics and Anti-inflammatory drugs	21,328,188.30	25,550,924.38	23,234,426.60
(3)	CNS drugs	30,746,197.68	23421524.62	30,264,897.30
(4)	Gastrointestinal medicines	4,525,398.72	5,598,815.09	5,183,502.69
(5)	Cardiovascular drugs	42,455,458.59	46,506,328.51	47,242,380.52
(6)	Anti-infective medicines, Antibacterials	50,513,836.84	68,207,175.34	77,644,113.59
(7)	Other Anti-infective medicines	18,321,053.66	23,951,291.04	36,383,120.25
(8)	Antineoplastic and immunosuppressive drugs	72,602,978.04	65,927,817.10	72,392,977.51
(9)	Antidotes and Endocrine drugs	16,926,707.86	15,209,988.91	19,039,929.92
(10)	Topical dermatological preparations	2,994,599.83	1,971,844.96	2,673,963.50
(11)	ENT preparations	2,208,031.00	2,456,214.04	2,000,968.50
(12)	Respiratory tract drugs	10,476,915.40	6,661,613.78	10,381,563.32
(13)	Vitamins and Minerals	6,880,499.45	5,115,727.65	6,054,840.00
(14)	Disinfectants and Antiseptics	9,865,037.60	8,891,550.25	9,871,303.56
(15)	Plasma substitutes and Parenteral Nutrition	52,725,902.00	55,633,309.13	38,653,487.20
(16)	Miscellaneous	14,598,700.00	9,549,277.32	5,873,793.00
(17)	Immunologicals	5,265,959.70	6,948,920.00	17,840,256.00
(18)	Oxytocics and Antioxytocics	6,342,560.00	4,907,109.50	2,956,310.00
	Total	400,625,444.17	406,391,886.87	452,064,244.35


Medicines for patients with cancer and immunosupressive medicines were the highest expenditure across all 3 years accounting for 16–18% of total expenditure (Table 9), consistent with the morbidity patterns (Table 8).

## Discussion

### ABC-VEN

Class A medicines were the fewest yet they consumed the highest percentage of total drug expenditure over the 3 years while class B constituted on average 17% but consumed approximately 15% of the annual expenditure (Table 2). Classes A and B medicines, which were an average of 245 medicine types for the 3 years, consumed 95% of the total drug expenditure, with the remaining Class C medicines, despite being the majority of medicine types, consumed only 5% of total drug expenditure (Table 2).

As a consequence of this, medicines belonging to Class A require more stringent managerial control, including assessing the appropriateness of prescribing within finite budgets, which is similar to activities in Europe for new premium priced medicines (Godman et al., 2014a, 2015, 2016; Kastanioti et al., 2016; Mousnad et al., 2016; Jakupi et al., 2018), as well as accurate data to drive forecasting of future demand, building on examples in other countries (Wettermark et al., 2010; Eriksson et al., 2017). Alongside this, close and frequent checks on budgets and stock levels as well as judicious purchasing, issuing of and inspection of medicines prescribed. It is with Class A medicines that the hospital can make maximum savings with its budget if pertinent with a reduction in the use of less cost-effective medicines in this category (Holloway and Green, 2003; Kastanioti et al., 2016; Mousnad et al., 2016). This may mean looking at issues of disinvestment in some categories (Parkinson et al., 2015; Brett et al., 2017; Guerra-Junior et al., 2017). Class B medicines require moderate control by middle level managers, whereas Class C medicines require only minimum control measures for order and purchase, and these functions can be delegated to lower level managers if pertinent since these medicines account for only 5% of the total budget (Gupta et al., 2007; Mousnad et al., 2016). However, they represent an appreciable number of stock items with an appreciable proportion of Vital or Essential medicines (Table 5); consequently, they should be closely supervised for Vital and Essential medicines.

A similar study conducted among the Armed Forces Medical College Hospital in India reported that, out of the 201 different types of medicines procured, 6.77% (104 medicine types) consumed 70.03% of the annual drug expenditure comprising Group A, whilst Group C medicines constituted 73.95% (1136) of the different types of medicines consumed but only 5% of annual drug expenditure (Kumar and Chakravarty, 2015). Migbaru et al. (2009) in Ethiopia reported that in 2009, 24 medicine items (9.60%) consumed 81.2% of the total annual drug expenditure (Group A), 51 items (20.40%) consumed 13.3% of annual drug expenditure (Group B) and the remaining 175 items (70.00%) consumed only 5.50% of the total drug budget (Group C). In 2013, 32 items (12.17%) consumed 76.15% of annual pharmaceutical expenditure (Group A), 47 items (17.9%) consumed 15.6% of the annual drug budget (Group B), with the remaining 184 items (69.96%) accounting for only 8.26% of the total annual drug budget (Group C) (Migbaru et al., 2009). Junita and Sari in Indonesia reported similarly that from 336 different medicines, 26 medicines (7.74%) consumed 70.84% of the annual drug budget (Class A), 37 medicines (11.01%) consumed 19.23% of the annual drug budget (Class B), whilst the majority of items (273 medicine types – 81.25%) accounted for only 9.93% of the drug budget (Class C) (Migbaru et al., 2009; Junita and Sari, 2012). Kastanioti et al in their study in Greece also found that approximately 9% (30 medicines types) accounting for nearly 70% of annual pharmaceutical expenditure in 2013 (Class A), whilst only 11% of items were responsible for nearly 80% of cumulative pharmaceutical expenditure in 2014 (Kastanioti et al., 2016). Similalry in the Sudan National Health Insurance Fund programme, Mousnad et al found that only a small number of medicine items (*n* = 80, 16.98%) accounted for largest proportion of annual expenditure (70.19% – Class A), whereas a large number of items (*n* = 288, 61.15% – Class C) accounted for only 9.92% of total annual drug expenditure (Mousnad et al., 2016).

The VEN analysis of the KNH drug store revealed that the majority of medicines belonged to the Vital (V) and Essential (E) categories, indicating that expenditure in the hospital is aimed at serving the health care needs of the majority of the population, which is encouraging. There were a number of medicines (141) which were non-formulary and were classified into the V, E, and N categories based on WHO classification (Kiguba et al., 2017). Migbaru et al. (2009) found that the majority of medicines in their hospital were also either Vital or Essential, and they also had the highest expenditure. A study performed at B. J. Government and Medical College, and Sassoon General Hospitals, analyzing a smaller number of different medicines, also showed that Vital medicines represented 148 (50.9%) of different medicines procured, Essential medicines 40.2% and Non-essential medicines at only 8.9% of total medicine items procured (Poorwa et al., 2013). This contrasts with a study conducted at the Armed Forces Medical College Hospital in India reporting that Vital medicines accounting for 13.14%, Essential drugs (E) for 56.37% and Non-essential medicines for 30.49% of the 1536 different medicines types in the hospital (ATC Level 5) (WHO, 2002). Devnani et al. (2010) also had similar findings with V medicines accounting for 12.11% of procured medicines types, E at 59.38% and N at 28.51%. Mousnad et al. (2016) also found a small number of items (*n* = 11, 2.34%) of Class V medicines accounted for 5.46% of the total drug budget whereas Class N medicines consisting of 45.01% items accounted for 26.43% of total annual expenditure (Mousnad et al., 2016).

Medicines belonging to the Vital category require continuous availability and reasonable safety stock, whilst Essential medicines require reduced stock levels, and non-essential medicines minimum managerial control over their availability and stocks (Devnani et al., 2010). This will be followed up in our hospital. In addition, non-formulary medicines should henceforth be considered by the Hospital DTC for possible inclusion into the Hospital formulary as they consumed only 2.4% of the total hospital expenditure on medicines during the 3 years and were considered necessary, and we will be following this up.

Our study had comparable results to the study of Kumar and Chakravarty (2015) which showed that 21, 51.17, and 27.83% of the medicines belonged to Category I, II, and III, respectively. Devnani et al. (2010) also reported that 22.09, 54.63, and 23.28% of the medicines procured were found to belong to category I, II, and III, respectively, accounting for 74.21, 22.23, and 3.56%, respectively, of annual drug expenditure. Medicines belonging to Category I require constant attention to their utilization and stocks; consequently requiring more selective control through Hospital DTCs and other measures (Bjorkhem-Bergman et al., 2013; Lima-Dellamora Eda et al., 2014; Matlala et al., 2017). The majority of medicines in KNH belong to Category II (47.7% of total medicine types), are of intermediate value (14% of total annual expenditure) (Table 6) and are an essential part of patient care; consequently they also require control and close supervision by middle level managers in the hospital.

Medicines belonging to the CE category, which are inexpensive and essential but account for just over 35.4% of the total medicine types and only on average 2.8% of total annual drug expenditure (Table 5), should be availed at all times. Category III (CN) consisting on average 21.3% of medicine types, but just over 1.1% of the total hospital budget, can be ordered in bulk to save on ordering costs if there is space available and they require minimum supervision.

Not surprisingly given the cost of medicines for patients with cancer and the extent of patients with infection in hospitals in Kenya (Atieno et al., 2018; Okoth et al., 2018), antineoplastic medicines and antibiotics consumed the highest amount of annual drug expenditure (Figure 1 and Table 9). This could be, as mentioned, that most antineoplastic medicines are expensive and KNH is currently the only referral hospitals that handles most of the cancer cases in Kenya (Atieno et al., 2018). Antibiotics are often highly prescribed, especially in view of high prevalence of infectious diseases in Kenya including HIV (Okoth et al., 2018), and since KNH is a referral hospital it stocks most of the expensive antibiotics including third line carbapenems.

### Therapeutic Category and Morbidity Patterns

The difference between the number of cases and drug expenditure in ICD-10 classes A00-B99, C00-D48, and D50-D59 (Table 8 and Figure 1) could be attributed to irrational use of medicines for the management of these patients. Alternatively, the medicines used to manage these patients could be expensive as seen potentially for treatments for patients with cancer (C00-D48) and infectious diseases (A00-B99); however, potentially necessary to improve outcomes as well as reduce morbidity and length of stay. Medicines for neoplasms consumed an average of 19% of the annual drug budget, and had the majority of the medicines in Class A. For ICD-10 classes I00-I99 (Diseases of the circulatory system), J00-J99 (Diseases of the respiratory system), and K00-K95 (Diseases of the digestive system), there also appears to be a mismatch between drug expenditure and the number of cases. This mismatch could be attributed to the use of inexpensive medicines to manage these cases; alternatively, there is a lack of enough medicines in the formulary to manage these patients effectively. Consequently, there is need for further studies on the reasons for the variability in expenditure and morbidity following this analysis, and this will be the subject of future research projects. However, we are mindful that the classification of diseases may not be that accurate (Holloway and Green, 2003), although this is not always the case among sub-Saharan countries (Mashalla et al., 2017).

The majority of medicines used to treat the most common diseases as per morbidity patterns are in Class A, and include injectables such as acyclovir, meropenem, heparin, enoxaparin, and sodium chloride infusion (Table 3). These medicines are either Vital or Essential and should be available at all times. Most of the medicines for patients with cancer are expensive but seen as essential; consequently, care should be taken to provide them. However, care when considering new treatments for patients with cancer given the limited health gain with the majority of new cancer medicines alongside their high prices (Kantarjian et al., 2013; Cohen, 2017; Godman et al., 2017b) potentially further increasing unaffordability (Atieno et al., 2018).

Theater medicines also accounted for considerable expenditure over the 3 years (Table 9); however, data on the number of cases operated per year is not currently available. This though will be looked at further in the future to see if any savings can be made without compromising care. The increase in expenditure for anti-infective and respiratory tract medicines over the years (Table 9) could be attributed to an increase in the number of cases for the two diseases over the years. Again this will be looked at further in light of our findings.

Injuries including burns are still the third most common cause of morbidity and mortality in Kenya, and a leading cause of death in other LMICs (WHO, 2017a). This is consistent with our data and other reports showing that KNH, being a public referral hospital, handles the majority of patients with severe injuries and burns in Kenya (Botchey et al., 2017; Myers et al., 2017). These conditions require long term costly treatment; consequently, most poor patients are referred to KNH where a single ward can incorporate as many as 100 patients.

Despite being the leading cause of morbidity at KNH, injuries and burns (5698 cases) accounted for only 3.1% of annual drug expenditure compared to infectious and parasitic diseases (3969 cases) that had the highest annual drug expenditure at 26.1%. The mismatch between the number of cases and drug expenditure for injuries and burns reported in this study may be due to the fact that expensive surgical dressings and other technologies for these conditions were excluded from this study because at KNH they are not purchased directly under the Pharmacy drug budget. This will be investigated further.

We are aware of a number of limitations with the data including incomplete and partial data including medicine prices. There were also incorrect entries and lost data in the health information systems for the morbidity data and some medicines are used to treat more than one disease. We also only considered the acquisition cost of medicines without looking further at their potential role in for instance reducing hospital length of stay. In addition, we have only carried out this study at one hospital, albeit the leading hospital in Kenya typically treating patients with cancer across Kenya and a referral hospital for patients with infectious diseases. However, despite this, we believe the findings give a comprehensive picture of current medicine utilization and expenditure within KNH providing direction not only to the senior management in KNH but to other hospitals in Kenya for the future.

## Conclusion

ABC analysis showed that whilst Class A medicines represented an average of only 13.7% of total medicine types procured they accounted for on average approximately 80% of total drug expenditure over the 3 years of study. Class C medicines, whilst being the majority, accounted for only 5% of total drug expenditure. VEN analysis showed that Vital and Essential medicines consumed approximately 90–95% of total drug expenditure over the 3 years with the remaining Non-essential medicines consuming only 5–11% of total drug expenditure. The ABC-VEN analysis showed further that items in Category I (AV, AE, AN, BV, and CV) consumed on average 85% of total annual drug expenditure, and should be the major focus for assessing their relative cost-effectiveness with increasing resource pressures as well as looking to eliminate any out-of-stock situations. Consequently, we believe the ABC-VEN techniques need to be adopted routinely by the Hospital Management in this and other similar hospitals throughout Kenya to ensure the optimal use of available resources for the patients. This is being followed up to maximize patient outcomes with available resources.

We are also aware that 20% of the medicines included in this study were non-formulary. This will again be looked at further for future inclusion in the hospital formulary such as Human normal immunoglobulin 5%; alternatively, restrictions placed on their use.

The TC and morbidity pattern data revealed a mismatch between drug expenditure and the number of cases seen in the hospital, which needs further investigation. We believe, based on our findings, that such analyses should also be routinely performed to help hospital management teams across Kenya address gaps between expenditure and the number of cases in the hospital. As a result, optimize their use of limited resources. This is now being followed up in our hospital, and we hope our study stimulates similar research in other hospitals in Kenya as well as other African countries. DTCs in Kenya and other sub-Sahara African countries should also focus on expenditures for antibiotics and antineoplastic medicines as these have been shown to be a significant proportion of hospital drug expenditure, which is likely to continue.

## Author Contributions

MO developed the design for the study. PK undertook the initial analysis with the support of MM, CO, and MO. PK together with MO, MM, and CO developed the initial manuscript including the analysis. AF, AK, and BG reviewed the data and contributed to the initial manuscript. All authors contributed to the final manuscript and approved the submitted version.

## Conflict of Interest Statement

The authors declare that the research was conducted in the absence of any commercial or financial relationships that could be construed as a potential conflict of interest.

## Footnotes

1 www.centralbank.go.ke/forex/
